# Abnormal phase entrainment of low- and high-gamma-band auditory steady-state responses in schizophrenia

**DOI:** 10.3389/fnins.2023.1277733

**Published:** 2023-10-24

**Authors:** Shoichiro Nakanishi, Shunsuke Tamura, Shogo Hirano, Junichi Takahashi, Kazutoshi Kitajima, Yoshifumi Takai, Takako Mitsudo, Osamu Togao, Tomohiro Nakao, Toshiaki Onitsuka, Yoji Hirano

**Affiliations:** ^1^Department of Neuropsychiatry, Graduate School of Medical Sciences, Kyushu University, Fukuoka, Japan; ^2^Department of Psychiatry, Division of Clinical Neuroscience, Faculty of Medicine, University of Miyazaki, Miyazaki, Japan; ^3^Department of Molecular Imaging and Diagnosis, Graduate School of Medical Sciences, Kyushu University, Fukuoka, Japan; ^4^National Hospital Organization Sakakibara Hospital, Mie, Japan; ^5^Institute of Industrial Science, The University of Tokyo, Tokyo, Japan

**Keywords:** auditory steady-state response (ASSR), evoked power, gamma oscillation, magnetoencephalography (MEG), phase locking angle (PLA), phase locking factor (PLF), schizophrenia

## Abstract

**Introduction:**

Gamma-band oscillatory deficits have attracted considerable attention as promising biomarkers of schizophrenia (SZ). Notably, a reduced auditory steady-state response (ASSR) in the low gamma band (40 Hz) is widely recognized as a robust finding among SZ patients. However, a comprehensive investigation into the potential utility of the high-gamma-band ASSR in detecting altered neural oscillations in SZ has not yet been conducted.

**Methods:**

The present study aimed to assess the ASSR using magnetoencephalography (MEG) data obtained during steady-state stimuli at frequencies of 20, 30, 40, and 80 Hz from 23 SZ patients and 21 healthy controls (HCs). To evaluate the ASSR, we examined the evoked power and phase-locking factor (PLF) in the time-frequency domain for both the primary and secondary auditory cortices. Furthermore, we calculated the phase-locking angle (PLA) to examine oscillatory phase lead or delay in SZ patients. Taking advantage of the high spatial resolution of MEG, we also focused on the hemispheric laterality of low- and high-gamma-band ASSR deficits in SZ.

**Results:**

We found abnormal phase delay in the 40 Hz ASSR within the bilateral auditory cortex of SZ patients. Regarding the 80 Hz ASSR, our investigation identified an aberrant phase lead in the left secondary auditory cortex in SZ, accompanied by reduced evoked power in both auditory cortices.

**Discussion:**

Given that abnormal phase lead on 80 Hz ASSR exhibited the highest discriminative power between HC and SZ, we propose that the examination of PLA in the 80 Hz ASSR holds significant promise as a robust candidate for identifying neurophysiological endophenotypes associated with SZ. Furthermore, the left-hemisphere phase lead observed in the deficits of 80 Hz PLA aligns with numerous prior studies, which have consistently proposed that SZ is characterized by left-lateralized brain dysfunctions.

## 1. Introduction

Gamma-band (30–100 Hz) oscillatory deficits have attracted considerable attention as a promising neurophysiological endophenotype of schizophrenia (SZ) due to their well-established association with various psychiatric symptoms and specific cognitive deficits observed in SZ (Uhlhaas and Singer, 2010; Hirano and Uhlhaas, 2021; Koshiyama et al., 2022; Onitsuka et al., 2022a,b,c; Sohal, 2022). Gamma-band oscillations are responsible for the temporal coordination of narrowly localized neural circuits throughout the brain (von Stein and Sarnthein, 2000; Fries et al., 2007; Buzsáki and Wang, 2012; Arnulfo et al., 2015; Buzsáki and Schomburg, 2015). In addition, they are well known to be generated by an appropriate balance between excitatory (E) and inhibitory (I) neurons, in which the excitatory output from pyramidal cells is precisely inhibited by fast-spiking inhibitory interneurons, such as parvalbumin (PV +) and somatostatin (SOM +) interneurons (Bartos et al., 2007; Sohal et al., 2009; Buzsáki and Wang, 2012). A prominent hypothesis concerning the pathophysiology of SZ proposes that *N*-methyl-D-aspartate receptors (NMDARs) on PV + neurons exhibit hypofunctionality, leading to diminished GABAergic signaling, excessive excitation of neurons, and subsequent deficits in gamma-band oscillations (Burgos and Lewis, 2008). Therefore, gamma-band oscillatory deficits are expected to serve as a potential biomarker related to altered E/I balance within the neural circuits in SZ (Gandal et al., 2012).

The auditory steady-state response (ASSR), which refers to the synchronized activity elicited by a click train or by amplitude-modulated sound, is frequently employed to evaluate gamma-band oscillatory deficits in SZ. A recent comprehensive review and meta-analysis investigating gamma-band ASSR deficits in SZ patients suggests that impairments in 40 Hz ASSR power and phase locking are pronounced and could serve as valuable probes for assessing neural circuit dysfunctions (Thuné et al., 2016; Onitsuka et al., 2022c). Numerous electroencephalography (EEG) studies have shown the deterioration of the gamma-band evoked power and phase-locking factor (PLF) (the degree of phase-locking evoked by stimuli) on 40 Hz ASSR, not only in chronic SZ patients (e.g., Kwon et al., 1999; Spencer et al., 2009; Kirihara et al., 2012) but also in first-episode SZ patients (e.g., Spencer et al., 2008; Tada et al., 2016) and subjects at clinical high risk for psychosis (Tada et al., 2016; Koshiyama et al., 2018). These recent findings provide compelling evidence that altered 40 Hz ASSR could serve as a neurophysiological marker with potential utility for early detection of psychosis.

Due to its remarkable temporal acuity and exceptional spatial resolution, magnetoencephalography (MEG) demonstrates remarkable proficiency in elucidating intricate neural dynamics (Hironaga et al., 2020; Tagawa et al., 2022). Several previous studies using MEG attempted to identify the brain regions responsible for gamma-band ASSR deficits in SZ (Hamm et al., 2011; Edgar et al., 2014, 2018; Murphy et al., 2020; Grent-‘t-Jong et al., 2021). Although most of these studies reported reduced evoked power or PLF on 40 Hz ASSR in the auditory cortex, Grent-‘t-Jong et al. (2021) found that subjects at clinical high risk for psychosis and patients with first-episode psychosis exhibited impaired 40 Hz ASSR power in subcortical regions such as the right thalamus and hippocampus. Likewise, leveraging the high spatial resolution of MEG, numerous studies have consistently reported a reduction in the laterality of the gamma-band ASSR in SZ (Teale et al., 2003, 2008; Maharajh et al., 2010; Tsuchimoto et al., 2011). Specifically, SZ patients demonstrated a weaker right hemisphere advantage in terms of 40-Hz ASSR evoked power and PLF when compared to healthy controls (HCs), indicating that SZ may be characterized by an abnormal hemispheric asymmetry in the 40-Hz ASSR.

Several recent studies focused on the gamma-band phase delay of the 40 Hz ASSR in SZ (Roach et al., 2019a,b, 2022; Yanagi et al., 2022). Notably, these studies revealed that gamma-band phase delay exhibited higher differential sensitivity to SZ (Roach et al., 2019b) and superior test–retest reliability (Roach et al., 2019a) compared to evoked power and PLF. In addition, Roach et al. (2022) identified a left-lateralized gamma-band delay in SZ by estimating 40 Hz ASSR within the bilateral auditory cortices using EEG data. However, EEG lacks adequate spatial resolution, necessitating a reassessment of the laterality pattern of gamma-band phase delay in SZ using MEG, which offers better spatial resolution than EEG. In addition to the examination of 40 Hz ASSR, it may be worthwhile to consider the high-gamma-band (80 Hz) ASSR as a possible neurophysiological marker for SZ (Onitsuka et al., 2022c). A limited number of studies (Hamm et al., 2011; Tsuchimoto et al., 2011; Parker et al., 2019) have revealed that patients with SZ exhibit decreased evoked power and PLF not only in the 40 Hz range but also in the 80 Hz range of ASSRs. Notably, our previous MEG study (Tsuchimoto et al., 2011) showed that evoked power and PLF of the 80 Hz ASSR demonstrated greater differential sensitivity to SZ patients compared to those of the 20, 30, and 40 Hz ASSRs. Moreover, early auditory deficits within the auditory cortices are known to play a crucial role in the pathophysiology of SZ (Javitt and Sweet, 2015; Dondé et al., 2023). Hence, it would be of great interest to investigate whether SZ patients exhibit reduced evoked power and PLF and a phase delay in the 80 Hz ASSR within the primary and secondary auditory cortex.

**FIGURE 1:**
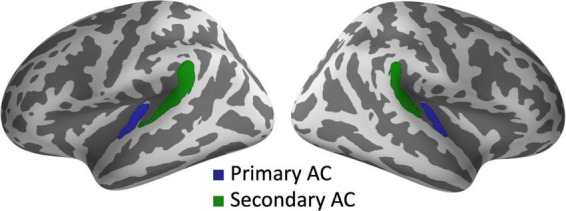
Regions of interest for source analysis of ASSRs. The green and blue areas indicate the primary and secondary auditory cortices, respectively, in both hemispheres. AC, auditory cortex.

The objective of the present MEG study is to investigate whether SZ patients exhibit abnormal phase entrainment, characterized by either a lead or delay, in both low- and high-gamma-band ASSRs within the auditory cortices. Additionally, we aimed to explore the laterality pattern of gamma-band phase entrainment (leads or delays) in SZ. We utilized MEG to measure ASSRs during 20, 30, 40, and 80 Hz click trains in both SZ patients and HCs. Our analysis focused on examining evoked power, PLF, and the degree of phase delay relative to the expected phase angle (phase-locking angle, PLA) in the bilateral primary (Heschl’s gyrus) and secondary auditory cortices (the planum temporale of the superior temporal gyrus) (Figure 1).

## 2. Materials and methods

### 2.1. Participants

Twenty-three SZ patients (10 males) and 21 HCs (10 males) participated in this study. All SZ patients were recruited from Kyushu University Hospital, and they were diagnosed based on the SCID-DSM V (First, 2002). The HC participants were recruited from the local community in Fukuoka City, and they and their first-degree relatives were free of Axis I psychiatric disorders. The exclusion criteria for both groups were as follows: (1) history of neurological illness or major head trauma that could result in abnormal electroencephalography results; (2) history of electroconvulsive therapy; (3) history of alcohol or drug dependence in their lifetime; (4) history of alcohol or drug abuse within the previous 5 years; or (5) a verbal intelligence quotient below 75. This study received approval from the Research Ethics Board of the Faculty of Medicine, Kyushu University (approval number 29038) and was carried out in accordance with the latest version of the Declaration of Helsinki. All the participants gave informed consent before the experiment.

Four SZ patients and 3 HC participants were excluded from data analysis because the MEG data were affected greatly by external magnetic noise (the exclusion criteria are stated in the description of MEG preprocessing). Finally, we employed the data of nineteen SZ patients (9 males with a mean age of 35.9 years) and 18 HCs (7 males with a mean age of 32.2 years). All participants were right-handed and did not have difficulty in listening to the stimuli used in this study. The symptoms of SZ patients were assessed by the Positive and Negative Syndrome Scale (PANSS) (Kay et al., 1987). Eighteen out of the 19 patients were receiving antipsychotics, with a mean daily dose equivalent to 499.12 mg of chlorpromazine (Inada and Inagaki, 2015). The mean illness duration in SZ patients was approximately 8.26 years. More detailed demographic and clinical characteristics are described in Table 1.

**TABLE 1:** Demographic and clinical characteristics of healthy control (HC) and schizophrenia (SZ) groups.

	Schizophrenia (*n* = 19)		Healthy control (*n* = 18)		*df*	*t* or χ^2^	*p*
	Mean	SD	Mean	SD			
Age (years)	35.9	10.9	32.2	14.1	32.9	−0.58	0.57
Sex (M/F)	9/10		7/11		1	0.04	0.85
SES	3.6	1	2.2	0.7	28.3	−4.55	<0.01
Parental SES	2.4	0.85	2.9	1.2	31.9	1.58	0.12
Education years	13.8	20.6	15.1	2	33.8	1.89	0.07
PANSS positive	19.6	7.2					
PANSS negative	23.2	8.8					
PANSS general	44.1	17.1					
Duration of illness (years)	8.3	8.9					
Medication dose (CPZ equiv., mg)	499.12	299.28					

The data are given as the mean ± standard deviation. Socioeconomic status (SES) and Positive and Negative Syndrome Scale (PANSS) data were not available for 1 and 3 patients, respectively.

### 2.2. Stimuli and apparatus

We used four types of click-train sounds with different train frequencies for the ASSR task. The 20, 30, 40, and 80 Hz click-train sounds with 500-ms duration were created using a 1-ms rectangle click sound. Each click-train sound was presented repetitively with an interstimulus interval of 500 ms until we obtained 200 trials with a peak amplitude less than amplitude criteria (gradiometer amplitude >3,000 fT/cm, magnetometer amplitude >3,000 fT) during online MEG recording. The order of employing the four frequencies was randomized across the participants. All stimuli were generated using a signal generator (Waveform Generator 7075, Hioki, Nagano, Japan) and presented to both ears of the participants through inserted earphones (ER-3A, Etymotic Research, IL, USA). The sound pressure level of these stimuli was set to 80 dB.

The MEG signals were acquired using a 306-channel system consisting of 204 planar-type gradiometers and 102 magnetometers (Neuromag, Elekta, Helsinki, Finland). The MEG signals were recorded at a sampling rate of 1 kHz with an online bandpass filter of 0.01–330 Hz. Since the magnetometers are sensitive to external noises, we used only the MEG signals of the gradiometers for data analysis. Before the recording, four head position indicator (HPI) coils were attached to the scalp, and the three fiducial points (the nasion and the left and right auricular points) of the head, the HPI positions, and approximately 200 head-surface points were measured using a three-dimensional digitizer. The accurate location of the head with regards to the sensor array was measured by the HPI coils.

T1-weighted structural images were obtained using a three-dimensional turbo field echo sequence using a 3T magnetic resonance imaging (MRI) scanner (Achieva TX, Philips, Best, Netherlands). The imaging parameters were as follows: repetition time = 8.2 ms, echo time = 3.8 ms, flip angle = 8°, field of view = 24 cm × 24 cm, number of echoes = 1, matrix = 240 × 240, inversion time = 1025.9 ms, number of slices = 190, and slice thickness = 1 mm. The MR images were aligned using the intercommissural line and the sagittal sulcus to correct head tilt. We constructed whole-brain source space from individual MRI data and created a boundary element model (BEM) of the brain using FreeSurfer 7.1.1. The coregistration of the MEG and MRI data was conducted based on the head position data obtained through the HPI coils and 3-D digitizer.

### 2.3. MEG pre-processing

We employed MNE-python 0.23.0^1^ and Python 3.9.6^2^ to perform MEG data analysis. To remove noise from MEG sensors, we first applied an oversampled temporal projection to the MEG data (Larson and Taulu, 2017). Second, we removed external noise from the MEG data and corrected head movements using the temporal extension of Signal-Space Separation (Nenonen et al., 2012). Third, we applied a high-pass filter of 1 Hz and a notch filter of 60 Hz and its harmonics (120, 180, 240, and 300 Hz) to MEG data. Finally, we removed artifacts caused by eye movement and electrocardiograms by applying an independent component analysis to the filtered data.

Using the artifact-free MEG data, we created epochs with 1500 ms duration, starting at 500 ms prior to stimulus onset and lasting for 1000 ms after stimulus-onset, separately for each stimulus condition (20, 30, 40, or 80 Hz). The epochs were rejected if the peak-to-peak amplitude of their waveform exceeded 5000 fT/cm. The four SZ patients and 3 HC participants were excluded from further analysis as described above because the number of epochs did not reach 200 in any of the four stimulus conditions. To avoid adverse effect of difference in the number of accepted epochs on time-frequency analysis results, 200 epochs selected from accepted epochs for each stimulus condition were submitted to further analysis. We also created evoked-subtracted epochs for each stimulus condition by subtracting an averaged evoked waveform from the waveform of each epoch.

### 2.4. Time-frequency analysis in source space

We calculated time-frequency representations (PLF, PLA, and evoked power) of ASSRs in the bilateral primary and secondary auditory cortices for each stimulus condition. The PLF measures the degree to which an oscillatory phase synchronizes across trials and ranges from 0 (random distribution) to 1 (perfect phase locking). The PLA is defined as the degree to which a certain participant’s oscillatory phase leads or lags relative to group (HC)-averaged phase angles of stimulus-evoked oscillations (Roach et al., 2019b). The evoked power is the spectrotemporal power of event-related activity. The primary and secondary auditory cortices are considered to correspond to the anterior transverse temporal gyrus and the planum temporale of the superior temporal gyrus, respectively, in the Destrieux atlas (Destrieux et al., 2010). Therefore, we defined these regions in both hemispheres as the regions of interest (ROIs) in this study (Figure 1).

The detailed procedure of source-level time-frequency analysis was as follows. First, we calculated a complex representation at each time-frequency point (time range from −500 to 1000 ms with 1 ms steps and frequency range from 10 to 100 Hz with 1 Hz steps) for each epoch or each evoked-subtracted epoch at each sensor by applying a Morlet wavelet transform (σ = 7.0) to the MEG signal. The norm of the obtained complex representation was changed to 1. The complex representations in source space were estimated from those in sensor space using noise-normalized minimum norm estimation (MNE), executed with dynamic statistical parametric mapping (dSPM) (Dale et al., 2000). For source estimation, we first constructed the forward solution, which models the propagation of the magnetic field from each brain region (mesh-patterned 8192 vertices were marked in the brain) to each sensor and was calculated based on the BEM of the brain. We next computed an inverse operator, which was used for estimation of the source-level complex representations from each epoch or each evoked-subtracted epoch, using the forward solution and a noise-covariance matrix calculated from pre-stimulus period (from –400 to –100 ms) signals of epochs or evoked-subtracted epochs.

For each vertex in source space, the averaged time-frequency complex representation across epochs was calculated. The norms of the averaged time-frequency complex representations were obtained as the time-frequency PLF. The time-frequency PLA was obtained for each vertex by re-expressing the angle of the averaged complex representation as its difference from an expected angle at each time-frequency point. According to Roach et al. (2019b), we defined a group-averaged angle of the HC group as the expected angle. To evaluate the time-frequency evoked power at each vertex on source space, we first obtained total and induced power values at each time-frequency point by calculating the averaged squared absolute values of the complex representations across epochs and evoked-subtracted epochs, respectively. The difference between the time-frequency total and induced powers was obtained as the time-frequency evoked power. The time-frequency PLF, PLA, and evoked power, which were obtained in each vertex, were averaged across vertices on each ROI. Regarding the time-frequency evoked power, a baseline correction was applied in each frequency point by subtracting a time-averaged evoked power in the pre-stimulus period (from –400 to –100 ms) from the evoked power at each time point.

### 2.5. Statistical analysis

The demographic variables [age, sex, socioeconomic status (SES), and education years] were compared between the HC and SZ groups using unpaired Welch’s *t*-tests or chi-square tests.

For statistical analysis of PLF and evoked power, we computed their mean values across time-frequency ranges, ranging from 30 to 530 ms and from 5 Hz below to 5 Hz above the train frequency for each stimulus condition. The obtained PLF and evoked power were subjected to three-way repeated-measures analyses of variance (rmANOVAs) with hemisphere (left/right) and ROI (first/secondary auditory cortex) as within-subjects factors and group (SZ/HC) as a between-subject factor separately for each stimulus condition (20, 30, 40, or 80 Hz).

We conducted a statistical analysis of the PLA following the previous study of Roach et al. (2019b). The time-averaged (200–400 ms) PLA at a frequency that was the same as the train frequency was obtained and converted to a z score based on the mean and standard deviation of the HC group for each stimulus condition, each hemisphere, and each ROI. Three-way rmANOVAs were performed for the z scored PLA value in the same way as statistical analyses of PLF and evoked power. The *post hoc* analyses were conducted for 40 and 80 Hz conditions as follows. For the 40 Hz condition, the unpaired *t*-test was conducted between HC and SZ groups on the averaged z-scale PLA across ROIs and hemispheres. Regarding the 80 Hz PLA, two-way rmANOVAs were conducted with group and hemisphere as factors for each ROI. In addition, unpaired *t*-tests were performed between the two groups for the z-scored PLA, separately for the left and right secondary auditory cortices.

The optimal sensitivity and specificity of gamma-band ASSR measures to differentiate between HC and SZ groups were determined via receiver operating characteristic (ROC) analysis to discuss a clinical applicability of these measures as a diagnostic or therapeutic tool in the future. Specifically, we conducted ROC analysis with each analysis index with a significant group difference (averaged evoked power across ROIs and hemispheres in the 80 Hz condition, averaged z-scale PLA across ROIs and hemispheres in the 40 Hz condition, or averaged z-scale PLA across ROIs in the 80 Hz condition) as a discriminant value. The ROC curve was drawn based on a trapezoidal rule. The effect size (Cohen’s d) was also calculated using the analysis indices used for the ROC analysis.

The relationships of gamma-band ASSR to clinical symptoms were examined using Spearman’s rank correlation analysis. We calculated Spearman’s correlations between the analysis indices with significant group differences and PANSS subscales (General, Positive, and Negative scales).

## 3. Results

### 3.1. Demographic data

There were no significant group differences in age (*t* = −0.58, *p* = 0.57) or sex (χ^2^ = 0.04, *p* = 0.85). The SES of the SZ group was significantly higher than that of the HC group (*t* = −4.55, *p* < 0.01), while there were no significant group differences in parental SES (*t* = 1.58, *p* = 0.12) or years of education (*t* = 1.89, *p* = 0.07).

### 3.2. PLF and evoked power

**FIGURE 2:**
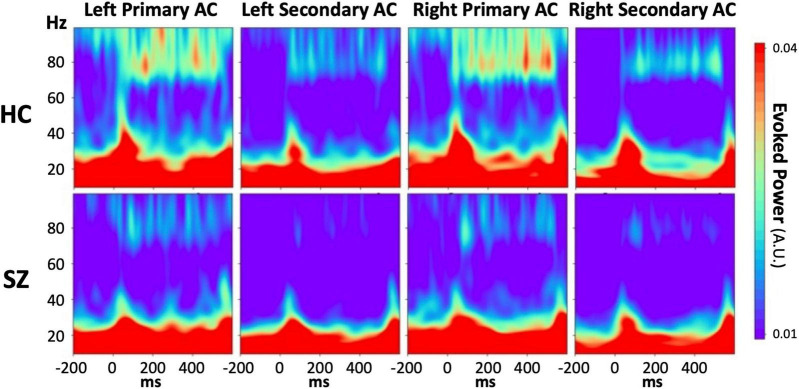
The grand average time-frequency maps of the evoked power during 80 Hz stimulation in each HC (**upper** line) or SZ (**lower** line) group for each region of interest. From left, the columns show the maps derived from the left primary auditory cortex, the left secondary auditory cortex, the right primary auditory cortex, and the right secondary auditory cortex. The color bar shows the values of the evoked power, in which red indicates a higher value and blue indicates a lower value. HC, healthy control; SZ, schizophrenia; AC, auditory cortex.

Supplementary Figures 1, 2 show group-averaged time-frequency PLFs and evoked powers, respectively, separately for each stimulus condition, each hemisphere, and each ROI. Regarding the PLF, we found neither a significant main effect of group nor group-related interactions in any stimulus condition, although the 80 Hz PLF was inclined to be lower in the SZ group than in the HC group. In terms of the evoked power, we found a significant reduction in 80 Hz evoked power in the SZ group (Figure 2). The three-way rmANOVA revealed a significant main effect of group (*F* = 4.42, *p* = 0.04) and no significant group-related interactions. We further conducted an unpaired *t*-test on the averaged evoked power values across ROIs and hemispheres as a *post hoc* analysis and confirmed that the evoked power during 80 Hz stimulation was significantly lower for the SZ group than for the HC group (*t* = −2.07, *p* = 0.049, Cohen’s *d* = 0.71) (Figure 2). While the evoked power at 20 and 40 Hz in the left primary auditory cortex showed a tendency to be higher and lower, respectively, in the SZ group than in the HC group, no significant main effect of group or group-related interactions was observed, except for the 80 Hz condition. The detailed results of the rmANOVAs on PLF and evoked power can be found in Supplementary Tables 1, 2, respectively.

### 3.3. PLA

**FIGURE 3:**
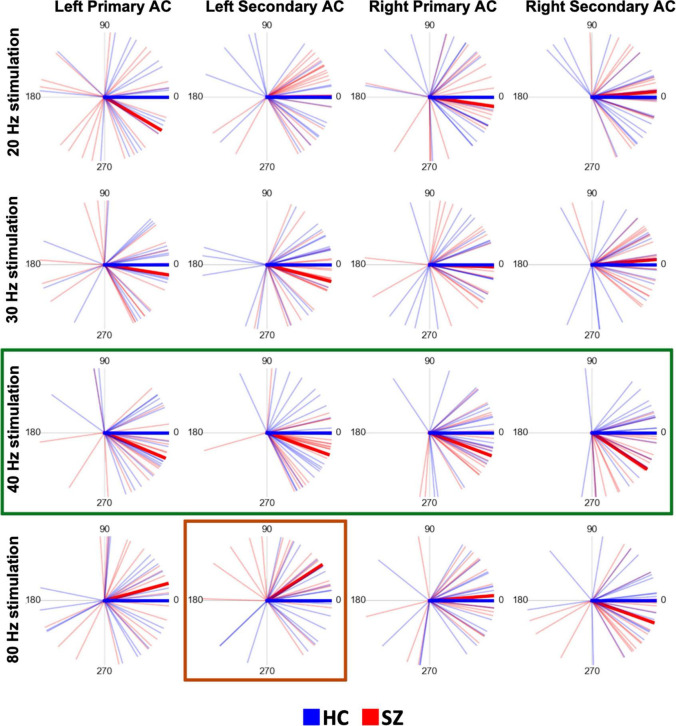
The time-averaged (200–400 ms) PLAs plotted on the polar coordinate of each subject and the grand average time-averaged PLA in each group (SZ or HC) at each stimulation frequency for each stimulus condition and each region of interest. In each plot, each thin red line indicates the time-averaged PLA of each SZ subject, each thin blue line indicates each HC subject, the thick red line indicates the grand average in the SZ group, and the thick blue line indicates the grand average in the HC group. The numbers placed circularly indicate angles (unit: degree). Each of the 4 plots on a horizontal line shows the plots derived at each stimulation frequency. Each column shows the plots in the same way as in Figure 2. In this figure, the thick green lines highlight the time-averaged PLAs at the 40 Hz stimulation, emphasizing that the SZ group had significant phase lags compared to the HC. The thick red lines highlight the time-averaged PLAs under 80 Hz stimulation in the left secondary auditory cortex, emphasizing that the SZ group had a significant phase lead compared to the HC group. HC, healthy control; SZ, schizophrenia; AC, auditory cortex; PLA, phase-locking angle.

Figure 3 shows the circular plots of individual PLAs at latencies of 200–400 ms separately for each stimulus condition, each hemisphere, and each ROI. The 40 Hz phase lags were clearly observed for the SZ group in both the primary and secondary auditory cortices of both hemispheres. The three-way rmANOVA on z-scored 40 Hz PLA showed that there was a significant main effect of group (*F* = 4.97, *p* = 0.03) with no group-related interactions. A *post-hoc t*-test confirmed that the SZ group had significantly lower values of z-scored 40 Hz PLA than the HC group (*t* = −2.21, *p* = 0.03, Cohen’s *d* = 0.75). On the other hand, the phase lead was observed on 80 Hz ASSR in the left secondary auditory cortex in the SZ group compared to HC. In the three-way rmANOVA on the z-scored 80 Hz PLA, we found a significant group × hemisphere × ROI interaction (*F* = 6.04, *p* = 0.02). The *post hoc* analyses revealed a significant interaction between group and hemisphere specifically in the secondary auditory cortex (*F* = 11.08, *p* < 0.01), while no significant interaction was observed in the primary auditory cortex. In the left secondary auditory cortex, the z-scored 80 Hz PLA was significantly higher in the SZ group than in the HC group (*t* = 3.04, *p* < 0.01, Cohen’s *d* = 1.03). Conversely, there was no significant group difference found in the right secondary auditory cortex (*t* = −1.54, *p* = 0.13). No main effect of group or group-related interactions was observed for the 20 Hz or 30 Hz conditions. The detailed results of the three-way rmANOVAs and the *post hoc* two-way rmANOVAs for the 80 Hz condition on the z-scored PLA are shown in Supplementary Table 3.

**FIGURE 4:**
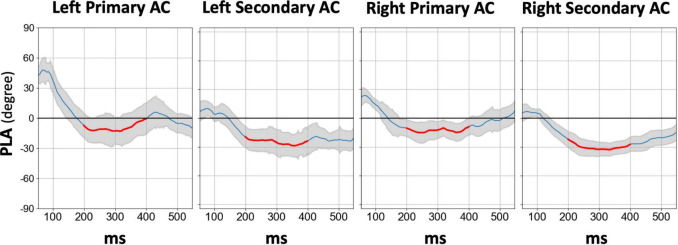
The grand average time course difference of 40 Hz PLA in the SZ group for each hemisphere and each ROI compared to HC, in which the PLA in SZ was subtracted from the PLA in HC. The thick red line in each time course indicates the time range used for statistical analysis of the PLA (200–400 ms). HC, healthy control; SZ, schizophrenia; AC, auditory cortex; PLA, phase-locking angle; ROI, regions of interest.

**FIGURE 5:**
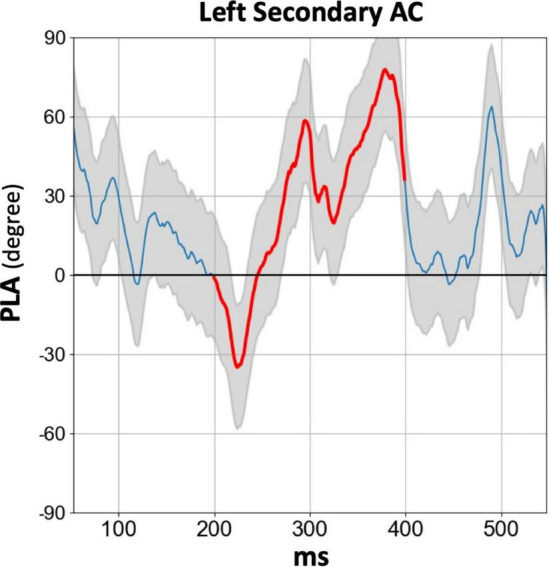
The grand average time course of 80 Hz PLA in the SZ group on the left secondary auditory cortex. The thick red line in each time course indicates the time range used for statistical analysis of the PLA (200–400 ms). HC, healthy control; SZ, schizophrenia; AC, auditory cortex; PLA, phase-locking angle.

Figure 4 displays the temporal profile of the 40 Hz PLA in the SZ group separately for each hemisphere and ROI. In the SZ group, we observed a phase delay starting at approximately 150 ms latency, persisting for over 200 ms across all hemispheres and ROIs. Conversely, a phase lead was observed at latencies preceding 100 ms, particularly in the primary auditory cortex. To further explore this, we conducted an additional statistical analysis focusing on the 30–100 ms PLA range. However, the three-way rmANOVA did not reveal any significant main effect of group or any group-related interactions (see Supplementary Table 4 for details). The time course of the 80 Hz PLA in the SZ group on the left secondary auditory cortex is also shown in Figure 5. Notably, the 80 Hz PLA in the SZ group showed abnormal phase lead throughout most of the ASSR stimulation, with occasional instances of phase delay around a latency of 200 ms.

### 3.4. ROC analysis

**FIGURE 6:**
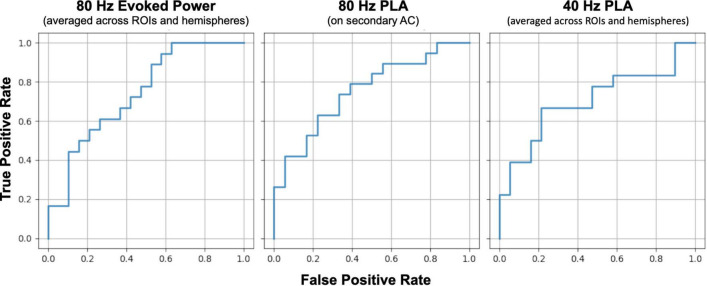
Comparison of receiver operating characteristic (ROC) curves between SZ and HC groups for 80 Hz evoked power **(left)**, 80 Hz phase lead **(middle)**, and 40 Hz phase delay **(right)**. HC, healthy control; SZ, schizophrenia; AC, auditory cortex; PLA, phase-locking angle; ROI, regions of interest.

We conducted ROC curve analyses to explore the discriminant value of ASSR measures. The left panel of Figure 6 shows the ROC curve for the averaged 80 Hz evoked power across ROIs and hemispheres, aiming to distinguish between the HC and SZ groups. The area under the curve (AUC) of this ROC curve was 0.74 [95% confidence interval (CI): 0.58–0.90]. The middle and right panel of Figure 6 shows the ROC curve for averaged z-scale 80 Hz PLA on the left secondary auditory cortex and averaged z-scale 40 Hz PLA across ROIs and hemispheres, respectively. The AUCs of the ROC curves for 80 Hz and 40 Hz PLA were 0.754 (95% CI: 0.60–0.91) and 0.705 (95% CI: 0.53–0.88), respectively. The obtained AUC values from 80 Hz evoked power, 40 Hz PLA, and 80 Hz PLA indicate acceptable discrimination between the HC and SZ groups (Hosmer et al., 2013).

### 3.5. Correlations of gamma-band ASSR measures with clinical symptoms

The 80 Hz evoked power was not significantly correlated with PANSS subscores (positive: rho = 0.34, *p* = 0.18; negative: rho = −0.08, *p* = 0.77; general: rho = −0.03 *p* = 0.93). Similarly, no significant associations were found between z-scored PLA and PANSS subscales for both 40 Hz (positive: rho = 0.43, *p* = 0.09; negative: rho = 0.11, *p* = 0.67; general: rho = 0.19, *p* = 0.46) and 80 Hz conditions (positive: rho = 0.06, *p* = 0.82, negative score: rho = −0.07, *p* = 0.79, general score: rho = −0.04, *p* = 0.88).

## 4. Discussion

The present study aimed to explore potential abnormalities in phase entrainment of low and high gamma-band ASSRs in patients with SZ, with a particular focus on hemispheric laterality. Our results revealed the presence of abnormal phase delay in the bilateral auditory cortices of SZ patients during the 40 Hz ASSR. In contrast, we observed an abnormal phase lead specifically localized to the left secondary auditory cortex during the 80 Hz ASSR, demonstrating higher discriminability between HC and SZ when compared to the other indices. Our findings suggest that left-lateralized deficits in the 80 Hz ASSR are a neurophysiological endophenotype of SZ.

### 4.1. Reduced 80 Hz evoked power in SZ patients

We detected reduced 80 Hz evoked power in SZ patients in source space by applying MNE-dSPM to MEG data, as with several previous MEG studies (Hamm et al., 2011; Tsuchimoto et al., 2011). In addition, we revealed that 80 Hz evoked power had higher discriminative power between the HC and SZ groups than 20, 30, or 40 Hz evoked power. Therefore, 80 Hz ASSR stimulation can be a robust tool to assess neural oscillatory deficits in SZ. High-gamma-band activity in the brain is considered to play critical roles in several cognitive functions, such as memory and language processing (e.g., Gaona et al., 2011; Kucewicz et al., 2014; Tamura and Hirano, 2023). Notably, our previous study (Tamura and Hirano, 2023) reported that high-gamma-band activity in the auditory cortex is closely involved with accurate processing of speech temporal information. Therefore, it is expected that assessing high-gamma-band activity in SZ patients would clarify the brain mechanisms underlying their cognitive dysfunctions, although we have to develop experimental paradigms requiring more cognitive demand compared to the ASSR paradigm in future studies. On the other hand, there was no group difference in 80 Hz PLF, although sensor-level analysis in our previous study revealed reduced evoked power and PLF at sensors surrounding auditory cortices in SZ patients (Tsuchimoto et al., 2011). One possibility is that this inconsistency might be attributed to the difference in cortical distributions between evoked power and PLF on 80 Hz ASSR. Specifically, an EEG study by Tada et al. (2021) reported that 80 Hz evoked power was localized to the auditory cortex in both hemispheres, while 80 Hz PLF was distributed in broad brain regions ranging from the temporal to frontal areas.

### 4.2. Phase delay of the 40 Hz ASSR in SZ patients

Similar to several previous studies (Roach et al., 2019a,b, 2022; Yanagi et al., 2022), we successfully confirmed the presence of a significant phase delay during 40 Hz ASSR in SZ patients. In addition, we observed that the 40 Hz PLA exhibited greater sensitivity to differentiate SZ compared to both 40 Hz evoked power and PLF. This result supports the utility of the PLA as a measure to detect gamma-band oscillatory deficits in SZ. When examining the time course of the 40 Hz PLA, we found that, when compared to the HC group, the SZ group showed a phase lead prior to approximately 150 ms, followed by a phase delay after a latency of approximately 150 ms. Notably, there were no significant group differences observed in the early latency phase lead. It is worth noting that the ASSR comprises two distinct components: a transient component (0–100 ms) and a sustained component (over 150 ms), which are believed to have distinct neural mechanisms (Ross et al., 2002; Ross and Pantev, 2004; Ding and Simon, 2009). Therefore, we propose that the observed 40 Hz phase lead and delay in the SZ group may indicate disruptions in both the transient and sustained components of 40 Hz ASSR.

However, we were unable to replicate the left-lateralized 40 Hz phase delay observed in a previous EEG study (Roach et al., 2022). In our study, we employed MEG with superior spatial resolution to EEG to assess the laterality of PLA deficits in gamma-band ASSRs. Notably, Edgar et al. (2017) practically confirmed that MEG has an advantage in detecting hemispheric laterality in 40 Hz ASSR compared to EEG. Our use of distributed source analysis (MNE-dSPM) is preferable to dipole source analysis, as gamma-band PLFs are known to originate from broad cortical sources (Tada et al., 2021). Therefore, it is plausible that our findings regarding the laterality of 40 Hz PLA deficits are more reliable than those of the previous study.

### 4.3. Left-lateralized phase lead of the 80 Hz ASSR in SZ patients

Patients with SZ exhibited an abnormal phase lead during the 200–400 ms latencies in the 80 Hz ASR, with a larger effect size compared to the phase delay observed in 40 Hz ASSR. This finding, in conjunction with the results of 80 Hz evoked power, underscores the value of utilizing 80 Hz ASSR stimulation. However, it is crucial to note that at these latencies, not only phase lead but also phase delay were observed in SZ. The phase angle in the high gamma-band ASSR might exhibit temporal instability, warranting further investigations to determine the optimal time range for assessing abnormal phase lead in SZ.

Furthermore, it is noteworthy that the abnormal phase lead observed in 80 Hz ASSR was specific to the left secondary auditory cortex. Consistent with our findings, there exists a substantial body of evidence from other studies demonstrating structural and functional abnormalities in the auditory cortex, particularly in the left hemisphere (Hajek et al., 1997; Kasai et al., 2003; Spencer et al., 2009; Hirano et al., 2010, 2015, 2020; Kompus et al., 2011; Kühn and Gallinat, 2012; Modinos et al., 2013; Shinn et al., 2013). This suggests that left-lateralized brain dysfunction might be a characteristic feature of SZ. Based on the hypothesis proposing that gamma-band oscillatory activity in the left auditory cortex plays a critical role in speech information processing with high temporal resolution (Poeppel, 2003; Poeppel et al., 2008), it is plausible that deficits in this activity could be linked to language dysfunction specific to SZ (Meyer et al., 2021). In future studies, given MEG’s remarkable temporal acuity and excellent spatial resolution, it would be intriguing to examine gamma-band PLA deficits focusing on subcortical areas such as the brainstem and thalamus because they were detected as signal sources of 80 Hz ASSR (Farahani et al., 2017, 2019).

### 4.4. Molecular mechanisms

As outlined in the Introduction, gamma-band oscillations are well known to reflect reciprocal interactions between excitatory and inhibitory neurons (E/I balance) (Bartos et al., 2007; Sohal et al., 2009; Buzsáki and Wang, 2012). Specifically, γ-aminobutyric acid (GABA)ergic and *N*-methyl-D-aspartate (NMDA) receptor antagonists have been shown to affect stimulus-phase-locked and non-phase-locked activities on gamma-band ASSRs (Vohs et al., 2012; Sullivan et al., 2015; Sivarao et al., 2016; Yamazaki et al., 2020). Nevertheless, the majority of preclinical studies have focused on exploring molecular mechanisms associated with reduced evoked power and PLF in the 40 Hz ASSR. As far as we know, there have been no studies focusing on high-gamma-band ASSR and its associated phase lead or delay. Therefore, further translational studies investigating high gamma-band activities are warranted.

### 4.5. Auditory dysfunctions in SZ

Among various perceptual and cognitive deficits, early auditory deficits play a crucial role in the pathophysiology of SZ (Javitt and Sweet, 2015; Dondé et al., 2023). A recent conceptual review (Dondé et al., 2023) highlights the utility of auditory measures for treatment targets and translational biomarker research. Alternation of gamma-band ASSR is one of the most prominent neurophysiological markers for early auditory deficits in SZ (Hirano and Uhlhaas, 2021; Onitsuka et al., 2022c). In addition, the gamma-band ASSR is well-known to reflect temporal processing ability in the auditory system (Baltus and Herrmann, 2015; Dimitrijevic et al., 2016). As described above, auditory-evoked high gamma-band activity in the left hemisphere is crucial in speech-temporal information processing (Tamura and Hirano, 2023). Thus, investigating auditory dysfunctions in SZ using the ASSR paradigm is useful for detecting impaired auditory temporal information processing in SZ.

### 4.6. Limitations

We obtained several meaningful findings concerning low- and high-gamma-band oscillatory dysfunctions in SZ patients; however, there are several limitations in the present study. First, the number of participants included in each group is relatively modest. Consequently, further studies with a larger sample size are warranted to validate the utility of 80 Hz ASSR stimulation and PLA measurement. Second, possibly due to the limited sample size, we were unable to observe reduced PLF and evoked power on 40 Hz ASSR in SZ patients. Third, during the MEG recording, the majority of patients were under antipsychotic medication, which leaves the possibility of antipsychotic influence on our reported results, although significant correlations of gamma-band ASSR measures with CPZ equivalent dose were not observed (40 Hz PLA: *rho* = 0.07, *p* = 0.79; 80 Hz evoked power: *rho* = −0.17, *p* = 0.50; 80 Hz PLA: *rho* = 0.06, *p* = 0.80). It is necessary to conduct additional research targeting untreated drug-free patients in future studies to evaluate 80 Hz ASSR and PLA with the effects of antipsychotic medication eliminated. Fourth, aberrant observations of the 80 Hz ASSR and PLA in SZ, as revealed from our investigation, currently lack clarity regarding their disease specificity. Consequently, prospective validation will necessitate the execution of large-scale cross-disease EEG or MEG investigations. Fifth, while our present study predominantly focused on analyzing the auditory cortex due to our implementation of a passive listening task that primarily engages early sensory functions, it would be crucial to investigate the ASSR in other areas by adapting tasks such as the attentional modulation paradigm that requires higher-order cognitive processes in future studies. Sixth, while assessing the dynamic time-course of PLA to detect abnormal entrainment is crucial, our technical constraints hindered us from employing a statistical approach that could retain temporal information within circular data. The emergence of analytical methods that take into account the dynamic time-course of PLA is anticipated. Last, we could not find a clear relationship between the gamma-band PLA measure and clinical symptoms, consistent with previous studies examining gamma-band PLA in SZ. We speculate that abnormal gamma-band phase lead or delay may serve as a trait marker rather than a state marker.

## 5. Conclusion

Considering the prominent phase-lead abnormality of the 80 Hz ASSR, which demonstrated the highest discriminative power between HC and SZ, we propose that a comprehensive investigation of PLA during the 80 Hz ASSR paradigm holds significant promise as a robust candidate for identifying neurophysiological endophenotypes associated with SZ. Moreover, the prominent left-hemisphere phase lead observed in the deficits of 80 Hz PLA aligns with numerous prior studies, which have consistently proposed that SZ is characterized by left-lateralized brain dysfunctions. To further explore whether this measure is a promising endophenotype related to the onset, the robustness of our findings ought to be confirmed through longitudinal investigations involving a larger sample size of first-episode SZ. Notably, to enhance the elucidation of its neural dynamics, the utilization of distributed source analysis alongside MEG data would facilitate the comprehensive exploration of gamma-band oscillatory functions across cerebral regions.

## Data availability statement

The datasets presented in this article are not readily available because the data that support the findings of this study are not openly available due to reasons of sensitivity and are available from the corresponding author upon reasonable request. Data are located in controlled access data storage at Kyushu University. Requests to access the datasets should be directed to YH, yhouji@gmail.com.

## Ethics statement

The studies involving humans were approved by the Research Ethics Board of the Faculty of Medicine, Kyushu University (approval number 29038). The studies were conducted in accordance with the local legislation and institutional requirements. The participants provided their written informed consent to participate in this study.

## Author contributions

SN: Data curation, Formal analysis, Investigation, Methodology, Visualization, Writing – original draft, Software. ST: Data curation, Formal analysis, Investigation, Methodology, Software, Visualization, Writing – original draft, Conceptualization, Resources, Supervision, Validation, Writing – review and editing. SH: Formal analysis, Resources, Supervision, Writing – review and editing, Project administration. JT: Writing – review and editing, Data curation, Investigation. KK: Data curation, Investigation, Writing – review and editing. YT: Data curation, Investigation, Writing – review and editing. TM: Data curation, Investigation, Writing – review and editing. OT: Data curation, Writing – review and editing, Methodology, Software, Supervision. TN: Supervision, Writing – review and editing, Funding acquisition, Resources. TO: Funding acquisition, Resources, Supervision, Writing – review and editing, Conceptualization, Methodology, Project administration. YH: Conceptualization, Funding acquisition, Methodology, Project administration, Resources, Supervision, Writing – review and editing, Data curation, Formal analysis, Investigation, Validation, Visualization, Writing – original draft.

## Footnotes

1
https://mne.tools/0.23/index.html

2
https://www.python.org/
