# Supplementary material for: Abnormal phase entrainment of low- and high-gamma-band auditory steady-state responses in schizophrenia
Source: Front Neurosci. 2023 Oct 24;17:1277733. doi: 10.3389/fnins.2023.1277733 (PMC10627971; doi:10.3389/fnins.2023.1277733)
Supplement: Supplementary file 1 [file Image_1.pdf]

Supplementary Figure 1.

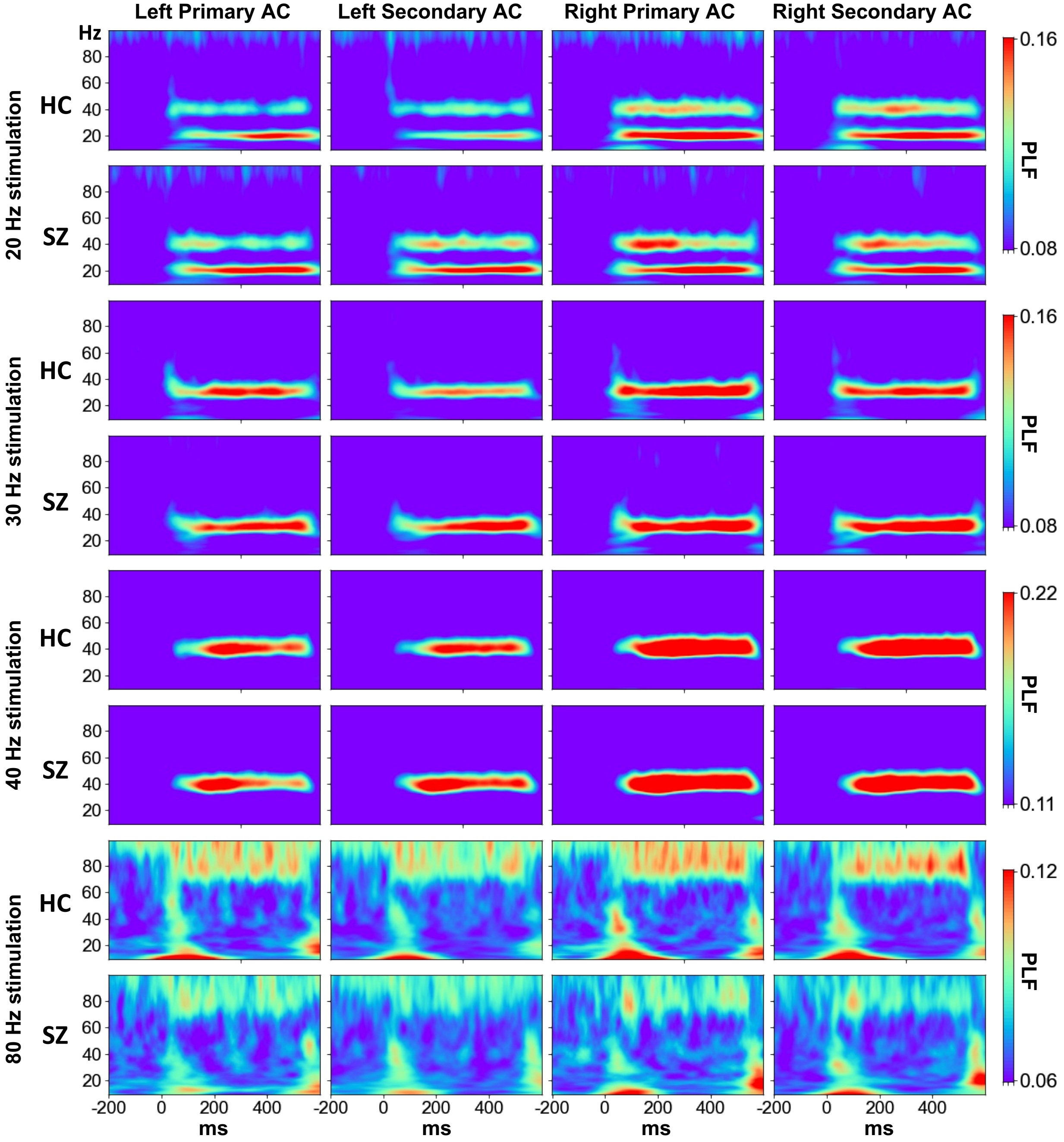

The grand average time-frequency maps of phase-locking factor (PLF) in each HC or SZ group at each stimulation frequency and each region of interest. Each line shows the plots derived at the combination of each stimulation frequency and each group, from the top, indicating the maps from 20 Hz stimulation and SZ group, 20 Hz and HC, 30 Hz and SZ, 30 Hz and HC, 40 Hz and SZ, 40 Hz and HC, 80 Hz and SZ, and 80 Hz and HC, respectively. Each column shows the maps in the same way as in **Figure 2**. Each color bar shows the values of PLF at each stimulation of frequency, in which red and blue color means the same in **Figure 2**.

HC: Healthy Controls, SZ: Schizophrenia, AC: Auditory Cortex
