# Supplementary material for: Abnormal phase entrainment of low- and high-gamma-band auditory steady-state responses in schizophrenia
Source: Front Neurosci. 2023 Oct 24;17:1277733. doi: 10.3389/fnins.2023.1277733 (PMC10627971; doi:10.3389/fnins.2023.1277733)
Supplement: Supplementary file 2 [file Image_2.pdf]

Supplementary Figure 2.

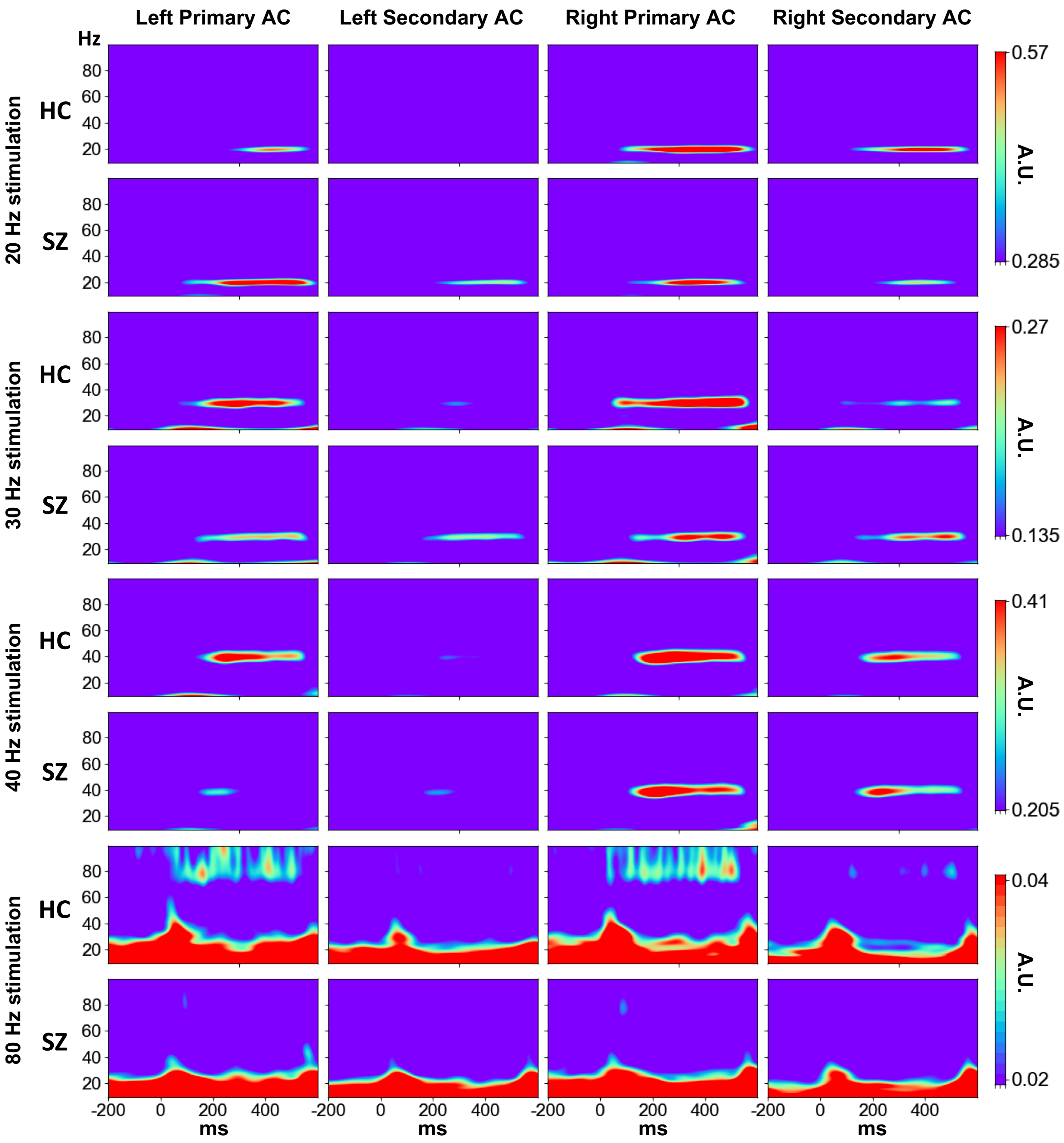

The grand average time-frequency maps of the evoked power in each HC or SZ group at each stimulation frequency and each region of interest. Each line and each column mean the same as in **Supplementary Figure 1**. Each color bar shows the values of the evoked power at each stimulation of frequency, in which red and blue color means the same in **Supplementary Figure 1**.
HC: Healthy Controls, SZ: Schizophrenia, AC: Auditory Cortex
