# Supplementary material for: Abnormal phase entrainment of low- and high-gamma-band auditory steady-state responses in schizophrenia
Source: Front Neurosci. 2023 Oct 24;17:1277733. doi: 10.3389/fnins.2023.1277733 (PMC10627971; doi:10.3389/fnins.2023.1277733)
Supplement: Supplementary file 3 [file Table_1.pdf]

Supplementary Table 1.
The detailed result of rmANOVA on PLF (ranging from 30 to 530 ms and from 5 Hz below to 5 Hz above the train frequency) for each stimulus condition.

20 Hz PLF source	*df*	*F* value	*p* value
Group	1	0.190	0.665
Hemisphere	1	2.27	0.141
Group × Hemisphere	1	1.68	0.204
Roi	1	2.57	0.118
Group × Roi	1	0.821	0.371
Hemisphere × Roi	1	0.0979	0.756
Group × Hemisphere × Roi	1	0.0202	0.888
30 Hz PLF source	*df*	*F* value	*p* value
Group	1	0.149	0.702
Hemisphere	1	3.65	0.0644
Group × Hemisphere	1	0.00120	0.973
Roi	1	0.435	0.514
Group × Roi	1	1.49	0.230
Hemisphere × Roi	1	1.20×10^{-4}	0.991
Group × Hemisphere × Roi	1	0.0135	0.908
40 Hz PLF source	*df*	*F* value	*p* value
Group	1	0.00451	0.947
Hemisphere	1	17.7	1.71×10^{-4}
Group × Hemisphere	1	0.238	0.629
Roi	1	0.0578	0.811
Group × Roi	1	0.001	0.972
Hemisphere × Roi	1	0.389	0.537
Group × Hemisphere × Roi	1	1.56	0.220
80 Hz PLF source	*df*	*F* value	*p* value
Group	1	2.27	0.141
Hemisphere	1	0.831	0.368
Group × Hemisphere	1	0.194	0.662
Roi	1	0.918	0.345
Group × Roi	1	0.341	0.563
Hemisphere × Roi	1	1.69	0.203
Group × Hemisphere × Roi	1	0.0342	0.854
