# Supplementary material for: Abnormal phase entrainment of low- and high-gamma-band auditory steady-state responses in schizophrenia
Source: Front Neurosci. 2023 Oct 24;17:1277733. doi: 10.3389/fnins.2023.1277733 (PMC10627971; doi:10.3389/fnins.2023.1277733)
Supplement: Supplementary file 4 [file Table_2.pdf]

Supplementary Table 2.
The detailed result of rmANOVA on evoked power (ranging from 30 to 530 ms and from 5 Hz below to 5 Hz above the train frequency) for each stimulus condition.

20 Hz evoked power source	*df*	*F* value	*p* value
Group	1	0.0110	0.917
Hemisphere	1	0.733	0.3.98
Group × Hemisphere	1	1.65	0.207
Roi	1	23.2	2.75×10^{-5}
Group × Roi	1	0.00309	0.956
Hemisphere × Roi	1	0.0560	0.815
Group × Hemisphere × Roi	1	0.818	0.372
30 Hz evoked power source	*df*	*F* value	*p* value
Group	1	0.0702	0.793
Hemisphere	1	1.77	0.192
Group × Hemisphere	1	0.191	0.665
Roi	1	7.28	0.0106
Group × Roi	1	3.42	0.0729
Hemisphere × Roi	1	0.252	0.619
Group × Hemisphere × Roi	1	0.271	0.606
40 Hz evoked power source	*df*	*F* value	*p* value
Group	1	0.285	0.597
Hemisphere	1	4.78	0.0356
Group × Hemisphere	1	0.218	0.643
Roi	1	6.59	0.0147
Group × Roi	1	1.40	0.245
Hemisphere × Roi	1	0.105	0.748
Group × Hemisphere × Roi	1	0.678	0.416
80 Hz evoked power source	*df*	*F* value	*p* value
Group	1	4.42	0.0429
Hemisphere	1	0.251	0.620
Group × Hemisphere	1	0.105	0.748
Roi	1	12.2	0.00133
Group × Roi	1	1.85	0.183
Hemisphere × Roi	1	0.467	0.499
Group × Hemisphere × Roi	1	0.0806	0.778
