# Supplementary material for: Abnormal phase entrainment of low- and high-gamma-band auditory steady-state responses in schizophrenia
Source: Front Neurosci. 2023 Oct 24;17:1277733. doi: 10.3389/fnins.2023.1277733 (PMC10627971; doi:10.3389/fnins.2023.1277733)
Supplement: Supplementary file 5 [file Table_3.pdf]

Supplementary Table 3.
The detailed rmANOVA on z-scored PLA for frequencies of 20, 30, 40, and 80 Hz for time periods of 200ms to 400ms.

20 Hz z-scored PLA	*df*	*F* value	*p* value
Group	1	0.934	0.341
Hemisphere	1	0.0110	0.917
Group × Hemisphere	1	0.0104	0.919
Roi	1	0.121	0.730
Group × Roi	1	0.114	0.737
Hemisphere × Roi	1	0.587	0.449
Group × Hemisphere × Roi	1	0.556	0.461
**30 Hz z-scored PLA**	*df*	*F* value	*p* value
Group	1	0.0326	0.858
Hemisphere	1	1.19	0.284
Group × Hemisphere	1	1.12	0.296
Roi	1	0.162	0.689
Group × Roi	1	0.153	0.698
Hemisphere × Roi	1	0.0162	0.900
Group × Hemisphere × Roi	1	0.0153	0.902
**40 Hz z-scored PLA**	*df*	*F* value	*p* value
Group	1	4.97	0.0322
Hemisphere	1	0.0117	0.914
Group × Hemisphere	1	0.0111	0.917
Roi	1	0.129	0.721
Group × Roi	1	0.122	0.729
Hemisphere × Roi	1	0.624	0.435
Group × Hemisphere × Roi	1	0.591	0.447
**80 Hz z-scored PLA**	*df*	*F* value	*p* value
Group	1	0.623	0.435
Hemisphere	1	5.73	0.0222
Group × Hemisphere	1	5.43	0.0257
Roi	1	0.611	0.440
Group × Roi	1	0.579	0.452
Hemisphere × Roi	1	6.37	0.0163
Group × Hemisphere × Roi	1	6.04	0.0191
