# Supplementary material for: Abnormal phase entrainment of low- and high-gamma-band auditory steady-state responses in schizophrenia
Source: Front Neurosci. 2023 Oct 24;17:1277733. doi: 10.3389/fnins.2023.1277733 (PMC10627971; doi:10.3389/fnins.2023.1277733)
Supplement: Supplementary file 6 [file Table_4.pdf]

Supplementary Table 4.
The detailed rmANOVA on z-scored PLA for frequencies of 20, 30, 40, and 80 Hz for time periods of 30ms to 100ms.

20 Hz z-scored PLA	*df*	*F* value	*p* value
Group	1	0.662	0.421
Hemisphere	1	0.0564	0.814
Group × Hemisphere	1	0.0534	0.819
Roi	1	0.740	0.396
Group × Roi	1	0.701	0.408
Hemisphere × Roi	1	0.950	0.336
Group × Hemisphere × Roi	1	0.900	0.349
**30 Hz z-scored PLA**	***df***	***F* value**	***p* value**
Group	1	0.402	0.530
Hemisphere	1	0.0847	0.773
Group × Hemisphere	1	0.0803	0.779
Roi	1	1.22	0.276
Group × Roi	1	1.16	0.289
Hemisphere × Roi	1	1.00	0.323
Group × Hemisphere × Roi	1	0.950	0.336
**40 Hz z-scored PLA**	***df***	***F* value**	***p* value**
Group	1	0.738	0.396
Hemisphere	1	0.00267	0.959
Group × Hemisphere	1	0.00253	0.960
Roi	1	0.694	0.410
Group × Roi	1	0.658	0.423
Hemisphere × Roi	1	0.0708	0.792
Group × Hemisphere × Roi	1	0.0671	0.797
**80 Hz z-scored PLA**	***df***	***F* value**	***p* value**
Group	1	0.0352	0.852
Hemisphere	1	4.01	0.0530
Group × Hemisphere	1	3.80	0.0593
Roi	1	0.0986	0.755
Group × Roi	1	0.0934	0.762
Hemisphere × Roi	1	3.20	0.0824
Group × Hemisphere × Roi	1	3.03	0.0906
